# Personalised profiling to identify clinically relevant changes in tremor due to multiple sclerosis

**DOI:** 10.1186/s12911-019-0881-1

**Published:** 2019-08-16

**Authors:** David G. Western, Simon A. Neild, Rosemary Jones, Angela Davies-Smith

**Affiliations:** 1 Department of Mechanical Engineering, University of Bristol, University Walk, Bristol, BS8 1TR UK; 2 Institute of Bio-Sensing Technology, University of the West of England, Coldharbour Lane, Bristol, BS16 1QY UK; 3 Department of Civil Engineering, University of Bristol, University Walk, Bristol, BS8 1TR UK; 4 MS Research Unit, Bristol & Avon Multiple Sclerosis (BrAMS) Centre, Southmead Hospital, Southmead Road, Bristol, BS10 5NB UK

**Keywords:** Biomedical signal processing, Movement analysis, Multiple sclerosis, Sensitivity and specificity, Tremor

## Abstract

**Background:**

There is growing interest in sensor-based assessment of upper limb tremor in multiple sclerosis and other movement disorders. However, previously such assessments have not been found to offer any improvement over conventional clinical observation in identifying clinically relevant changes in an individual’s tremor symptoms, due to poor test-retest repeatability.

**Method:**

We hypothesised that this barrier could be overcome by constructing a tremor change metric that is customised to each individual’s tremor characteristics, such that random variability can be distinguished from clinically relevant changes in symptoms. In a cohort of 24 people with tremor due to multiple sclerosis, the newly proposed metrics were compared against conventional clinical and sensor-based metrics. Each metric was evaluated based on Spearman rank correlation with two reference metrics extracted from the Fahn-Tolosa-Marin Tremor Rating Scale: a task-based measure of functional disability (FTMTRS B) and the subject’s self-assessment of the impact of tremor on their activities of daily living (FTMTRS C).

**Results:**

Unlike the conventional sensor-based and clinical metrics, the newly proposed ’change in scale’ metrics presented statistically significant correlations with changes in self-assessed impact of tremor (*max* *R*^2^>0.5,*p*<0.05 after correction for false discovery rate control). They also outperformed all other metrics in terms of correlations with changes in task-based functional performance (*R*^2^=0.25 vs. *R*^2^=0.15 for conventional clinical observation, both *p*<0.05).

**Conclusions:**

The proposed metrics achieve an elusive goal of sensor-based tremor assessment: improving on conventional visual observation in terms of sensitivity to change. Further refinement and evaluation of the proposed techniques is required, but our core findings imply that the main barrier to translational impact for this application can be overcome. Sensor-based tremor assessments may improve personalised treatment selection and the efficiency of clinical trials for new treatments by enabling greater standardisation and sensitivity to clinically relevant changes in symptoms.

## Background

### Introduction

Upper-limb tremor can arise as a symptom of various neurological pathologies including Parkinson’s disease, essential tremor, and multiple sclerosis (MS). Clinical assessments seek to determine the nature and severity of the tremor, in order to inform treatment selection. Due to technological developments, sensor-based assessments are increasingly being considered to improve precision, accuracy, and objectivity, quantifying tremor according to the frequency and amplitude of movement in the hand or individual joints [12–14, 25, 27, 30, 34].

The translational impact of this research has been limited by the fact that, despite the precision and sensitivity of available sensors, the recorded data exhibit considerable test-retest variability. This limitation was highlighted recently by a task force of the International Parkinson and Movement Disorder Society (MDS) [14]:

In most clinical studies, investigators are interested in changes in tremor amplitude or occurrence that exceed random variability… Currently, there is no evidence that the minimum detectable change is smaller when transducers are used.

The task force also noted that “there is considerable test-retest random variability in tremor amplitude”. One might perceive this as a fundamental limit on the clinical value of tremor amplitude measurements, leaving little advantage to be gained from the use of accurate sensors. However, in the present paper we demonstrate that a considered analysis of sensor-based measurements can minimise the need for blunt approximations, such as a population-wide test-retest variability or the reduction of tremor movements to a single axis. In particular, we present three key methodological proposals: Multiple axes: Tremor should be measured across multiple joints/degree-of-freedom to increase information capture. Previous authors have adopted this paradigm, with limited translational success; we assert that the following additional proposals are essential to ensure effective exploitation of the available information. Personal tremor profiling: Changes in tremor amplitude should be measured in a way that accounts for the individual’s ‘typical’ tremor, for improved differentiation between tremor changes and other confounding influences. Personalised variability control: From sensor-based assessments, some estimate of the ‘random’ variability of the subject’s tremor may readily be extracted. Such an estimate may be used in place of a population-wide estimate to infer the significance of any observed change.

In the present paper, we demonstrate that these proposals substantially improve the power of sensor-based tremor measurements to detect clinically relevant changes in symptoms, outperforming directly interpreted sensor-based measurements and the most widely used clinical tremor rating scale for MS. Here we take ‘clinically relevant changes in symptoms’ to mean those that yield measurable changes in functional ability or the perceived impact of the tremor.

Our method is demonstrated in a cohort of people with upper-limb tremor due to MS performing a standardised finger-to-nose task, the most commonly used task in the assessment of this MS symptom [20]. Currently, there are no universally effective treatments for MS tremor [20]. Estimates of the prevalence of tremor in MS vary from approximately 25 to 60 percent [20]. The prevalence of tremor in the registry of the North American Research Committee on MS (NARCOMS) is estimated to be at least 45 percent [28].

The assessment of tremor in MS is particularly challenging as it is often concomitant with ataxia or other less well-defined movement disorders, and the relative contribution of each to the observed movement may not be easily distinguished [20]. Intention tremor, defined as arising in target-directed movements [5], is a particularly disabling form that is especially prevalent in MS compared with other aetiologies. Due to these factors, the ‘tremors’ observed in MS are often less consistently periodic than those seen in other pathologies, although they still satisfy the Movement Disorder Society’s broad definition of tremor as “rhythmical, involuntary, oscillatory movement of a body part” [5]. The complexity and inconsistency of MS tremor amplitude exacerbates the test-retest variability noted by the MDS task force [14], thus degrading the statistical power of trials for new treatments. However, in our experience, the essential characteristics of tremor remain relatively static in each patient from one assessment to another, in that recognisable traits, such as the relative involvement of individual joints, persist across multiple observations.

### Prior work

The general task of objectively detecting changes in tremor symptoms may be considered as a sequence of four stages:

1. recording the movement with any suitable sensor type
2. extracting movement parameters such as joint angles or limb segment positions and orientations from raw data
3. extracting tremor features such as amplitude and frequency from the movement parameters
4. comparing tremor features between two or more recordings from the same subject

The main contribution of this paper is in the fourth stage of this process, a stage which has not been considered in detail in prior work.

A thorough discussion of suitable motion capture technologies is beyond the scope of this paper. They include optical tracking systems, markerless machine-vision based systems, on-body sensors, and any combination of the above. As an alternative to capturing the movement itself, electromyography recordings may be used to directly capture the underlying muscle activity [16].

In this paper, we use the term inertial measurement unit (IMU) to refer to any combination of triaxial accelerometers, gyroscopes, magnetometers and/or GPS sensors in a single wearable package. IMUs are widely used in recent tremor studies due to their combination of low-cost, high accuracy, and ease of use. Some IMU systems are now marketed specifically for tremor analysis, such as the Kinesia system [12] and others as reviewed by Ossig et al. [22]. IMUs, combined with sensor fusion algorithms, allow any rigid body part’s position and orientation to be captured in three dimensions. Furthermore, applying IMUs to multiple adjacent body parts allows the extraction of the movements of individual joints between those body parts. This approach can be a convenient means of capturing motion across entire limbs, or even the whole body, according to a simplified skeletal model.

Numerous studies have explored the use of this technology to investigate tremor and other movement disorders or to control intelligent orthotic/assistive devices [1, 2, 10, 21, 25, 30], and several studies have focussed on the development and assessment of signal processing techniques to reliably quantify tremor based on these recordings [11, 18, 24, 29].

The validity of sensor-based tremor measurements as a means of controlling therapeutic devices or quantifying tremor characteristics – stages 1–3 in the process outlined at the start of this section – is now well established. It has also been shown that sensor-based tremor assessments may be compared with a database of annotated recordings to usefully classify tremor type and severity in Parkinson’s disease and essential tremor [1, 4, 27, 31]. However, the works noted above do not address a crucial clinical need: the need to identify changes in symptoms within individuals in order to monitor disease progression and evaluate treatment efficacy. For these applications, the usefulness of detailed and accurate measurements is limited if the test-retest variability remains high. Given that the symptom itself is known to exhibit substantial intrinsic variability, it is unsurprising that the MDS task force [14] describe this as a key limitation of sensor-based tremor assessments. That may be so, but it is not necessarily an insurmountable one, as we sought to demonstrate in the present study.

### Hypothesis

The persistence of easily identifiable personal tremor characteristics across highly variable amplitude measurements suggests that a one-size-fits-all comparison of tremor features may not be appropriate to capture an individual’s changing state. Taking their signature features into account during the comparison may allow meaningful changes to be distinguished from random variability. To our knowledge, no prior work has been explicitly focussed on this fourth stage of the process of identifying changes in tremor symptoms.

We proposed to overcome the problem described above by exploiting the convenience offered by modern systems such as IMUs in capturing multiple movement parameters simultaneously. The high dimensionality of such data opens up the possibility of seeking mathematical transformations in which clinically significant changes and incidental ones are largely separated into orthogonal axes, essentially removing the latter from the former. We hypothesised that the inherent variability of MS upper-limb tremor, which inhibits the detection of clinically significant changes, can be overcome by separating the characteristic features of the movement from more ‘random’ disturbances like large amplitude deviations in the movement pathway typical of ataxic movement.

## Methodological proposal

Our methodological proposal addresses the question of how a detailed tremor dataset may be processed to characterise tremor cases in a manner that is robust to clinically irrelevant variation in the symptom. In general our approach assumes that multiple measurements are available from any given subject. Each measurement comprises tremor amplitude estimates extracted from multiple movement parameters (e.g. joint rotations). For comparability, all measurements are assumed to be from separate executions of the same task. At the core of our proposal is the collective analysis of a subject’s available measurements, leading to a personalised data transformation that exposes clinically distinct elements of the individual’s change in symptoms.

### Characteristic vectors

The transformations considered rely on the identification of a ‘characteristic vector’ that describes the subject’s archetypal tremor in a particular task. To generate a subject’s characteristic vector we first define, for each measurement, a measurement vector ***a***_***i***_ containing one tremor amplitude estimate (the form of which is discussed in “Extraction of tremor amplitudes” section) from each of the *N* available movement parameters. If no valid estimate is available for a given parameter, such as when tremor is not reliably detected in a particular joint, its place may be held by a null value. The subscript *i* represents the *i*^th^ measurement.

1 $$  \boldsymbol{a_{i}} = \left[\begin{array}{l} a_{i,1}, a_{i,2}, \hdots a_{i,N} \end{array}\right]^{T}  $$

For the *j*^th^ movement parameter considered, the corresponding element *a*_*c,j*_ of the subject’s characteristic vector is then calculated by summing *a*_*i,j*_ across *V*_*j*_, the set of indices of the measurements (task executions) from this subject containing valid results for the *j*^th^ parameter, as written below.

2 $$  a_{c,j} = \frac{\sum\nolimits_{i \in V_{j}}^{}{a_{i,j}}}{m_{j}}  $$

*m*_*j*_ is the number of valid measurements for the *j*^th^ parameter. If, for a given parameter, no valid amplitude estimates are available (i.e. *m*_*j*_=0), a null value was recorded for *a*_*c,j*_.

The full characteristic vector, ***a***_***c***_, represents a grouping of representative amplitude estimates from that subject in multiple joints or other movement parameters, and can be used to personalise the interpretation of any particular measurements from that subject. The notion that personalised profiling might be useful in tremor assessment has particularly strong physiological justification in the case of MS. The precise symptoms experienced by a person with MS are highly individual, due to the subject-specific manner in which the eponymous *sclerae*, or plaques, are distributed through the central nervous system.

### Personalised measures of change

In this section, we formally define our principal metric of interest, the change in scale of the tremor, along with its natural counterpart, the change in profile of the tremor.

Once a characteristic vector is established, the difference between any two measurement vectors can be expressed in terms of two new parameters: change in scale and change in profile.

#### Change in scale

**Fig. 1:**
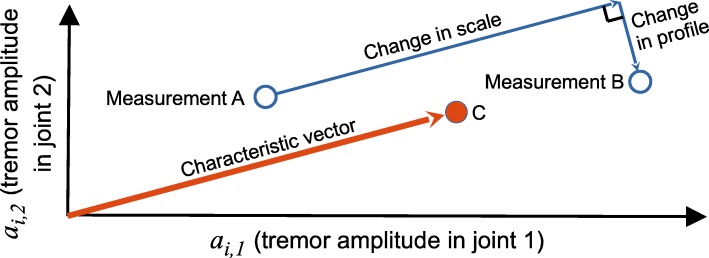
Conceptual illustration of the scale and profile changes in tremor between two measurements (A and B), applied to a hypothetical example using only two joint rotations as the basis parameters. The characteristic vector is defined by point C, whose coordinates are typical values of tremor in joints 1 and 2 for this limb (based on other measurements, not shown). Note that the change in scale is measured parallel to the characteristic vector, and the change in profile is measured perpendicular to it

As shown in Fig. 1 for the case with two movement parameters, a change in scale is measured parallel to the characteristic vector. The change in scale *d*_*S*_ between measurements A and B may be expressed as follows:

3 $$  \text{scale change metric:} \quad\quad d_{S}\left(\boldsymbol{a_{A}},\boldsymbol{a_{B}},\boldsymbol{a_{c}}\right) = \left(\boldsymbol{a_{B}}-\boldsymbol{a_{A}} \right) \cdot \boldsymbol{\hat{a}_{c}}  $$

where $\boldsymbol {\hat {a}_{c}}$ is the characteristic tremor vector for that limb, normalised to have unit magnitude. If some element *a*_*i,j*_ from any of ***a***_***A***_,***a***_***B***_, or $\boldsymbol {\hat {a}_{c}}$ contains a null value, the *j*^*th*^ parameter is excluded from all three vectors before calculating *d*_*S*_.

The design intent of the scale change metric is to give greater weighting to those joints in which the individual’s tremor is most consistently pronounced, thereby reducing ‘noisy’ contributions from elsewhere. Various adjustments may be considered to further pursue this intent, and two such strategies are considered within this study. The influence of lesser joints may be eliminated completely by removing the corresponding elements of ***a***_***A***_,***a***_***B***_, and $\boldsymbol {\hat {a}_{c}}$. Alternatively, the relative influence of the most tremulous joints may be increased by some tuning of the characteristic vector, such as by squaring each element thereof, as follows.

4 $$  \boldsymbol{a_{c2}} = \left[\begin{array}{l} a_{c,1}^{2}, a_{c,2}^{2}, \hdots a_{c,N}^{2} \end{array}\right]^{T}  $$

This tuned characteristic vector may be normalised to unit magnitude and employed in an alternative definition of the scale change metric, referred to henceforth as the scale2 change metric.

5 $$  \text{scale2 change metric:} \quad d_{S2}\left(\boldsymbol{a_{A}},\boldsymbol{a_{B}},\boldsymbol{a_{c}}\right) = \left(\boldsymbol{a_{B}}-\boldsymbol{a_{A}} \right) \cdot \boldsymbol{\hat{a}_{c2}}  $$

#### Change in profile

Any other change in the tremor vector is captured in the component perpendicular to the characteristic vector, and may be interpreted as a deviation from/towards the established characteristic blend, or profile, of the tremor. This difference measurement may be calculated as the magnitude of that perpendicular component, as follows.

6 $$  \text{profile change metric:} \quad d_{P}(\boldsymbol{a_{A}},\boldsymbol{a_{B}},\boldsymbol{a_{c}}) = \lvert (\boldsymbol{a_{B}}-\boldsymbol{a_{A}}) \times \boldsymbol{\hat{a}_{c}} \rvert  $$

Because changes in profile may manifest in any direction perpendicular to the characteristic vector, they cannot be interpreted as an increase or decrease; they are always positive by construction. Hence they cannot be used to detect whether symptoms have worsened or improved, though they may be useful in identifying when a subject’s typical tremor characteristics have altered significantly.

### Normalisation

Based on the preceding definitions, the movement parameters with the greatest amplitude will clearly have the greatest influence on the direction of ***a***_***c***_ and, consequently, *d*_*S*_ and *d*_*P*_. This weighting may be appropriate if all parameters are of comparable type (e.g. all are joint angles). However, if the parameters are heterogeneous (e.g. if they include displacement and rotation of the hand, which are measured in different units), such uneven weighting is not appropriate.

Measurements involving heterogeneous parameters must be normalised before a meaningful characteristic vector can be calculated. If a reasonable estimate of each parameter’s distribution across a given population is available, then each may be converted to a z-score by subtracting the population mean before dividing by the standard deviation.

### Controlling for intrinsic variability

The MDS task force [14] highlighted the need to distinguish meaningful amplitude changes from random variability. To embody this intent, we scale the metric of clinical interest (the average difference between visits) by an estimate of intrinsic variability (the average difference between measurements taken in the same visit). Thus the adjusted value of some arbitrary difference metric *d*_*X*_ between the days *p* and *q* is calculated as follows

7 $$  d_{X}'\left(p,q,\boldsymbol{a_{c}}\right) = \frac{\left\langle d_{X}\left(\boldsymbol{a_{p1}},\boldsymbol{a_{q1}},\boldsymbol{a_{c}}\right),d_{X}\left(\boldsymbol{a_{p2}},\boldsymbol{a_{q2}},\boldsymbol{a_{c}}\right) \right\rangle}{\left\langle d_{X}\left(\boldsymbol{a_{p1}},\boldsymbol{a_{p2}},\boldsymbol{a_{c}}\right),d_{X}\left(\boldsymbol{a_{q1}},\boldsymbol{a_{q2}},\boldsymbol{a_{c}}\right) \right\rangle}  $$

where ***a***_***p1***_ and ***a***_***p2***_ are the measurement vectors for the first and second recordings on day *p*, respectively, and likewise ***a***_***q1***_ and ***a***_***q2***_ for day *q*. Angle brackets denote the average of the enclosed elements.

## Method

We evaluated the proposed metrics using movement data recorded from the dominant arms of 24 people with MS tremor, 19 of whom provided recordings from that arm on a second visit. On each visit, the subject was also assessed according to the Fahn-Tolosa-Marin Tremor Rating Scale (FTMTRS) [8], explained in greater detail in “Metrics from a conventional clinical scale” section. No interventions were administered for the purposes of the study, and none of the subjects received a change in treatment of their tremor symptoms between visits. Natural changes in tremor symptoms were expected due to disease progression as well as daily factors such as weather and fatigue.

The characteristics of the cohort on each visit are summarised in Table 1. Student’s paired t-tests revealed no significant change in FTMTRS A or B (*p*=0.9 and *p*=0.8, respectively), but the increase in FTMTRS C was significant (*p*=0.002).

**Table 1:** Cohort Summary

			FTMTRS - Visit 1			FTMTRS - Visit 2			
Patient ID	M/F	age	A	B	C	A	B	C	Days
1	F	57	3	18	24	4	17	24	216
2	F	44	4	20	24				
3	M	52	2	5	8	1	5	11	246
4	F	57	2	5	14	2	6	19	194
5	F	34	2	6	20	2	6	19	222
6	M	60	4	20	24	4	20	24	209
7	F	44	3	18	18	2	13	23	192
8	F	45	3	19	20	2	10	24	381
9	F	49	0	0	9	0	0	10	206
10	M	42	1	13	18	1	20	24	155
11	F	48	1	0	5	1	1	5	189
12	F	46	2	12	19	1	9	19	101
13	F	43	1	2	8	1	1	9	267
14	M	33	1	5	13	1	5	14	225
15	F	55	1	3	21				
16	F	48	1	2	16	3	12	21	154
17	M	50	0	1	11	1	3	13	138
18	M	31	2	1	16	1	3	14	147
19	F	45	1	1	6	1	3	10	28
20	F	30	1	3	12	1	4	11	21
21	F	31	0	0	3	1	1	4	35
22	F	44	2	19	20	2	13	22	27
23	F	67	2	9	20	2	11	19	16
24	F	34	0	0	1	1	0	6	12
	mean	45	1.6	7.6	14.6	1.6	7.4	**15.7** ^**^	153.7
	min	30	0	0	1	0	0	4	12
	max	67	4	20	24	4	20	24	381

^**^Significant change from visit 1; *p*<0.01

### Recordings

In each visit, the subject performed two instances of a finger-to-nose task with each arm in accordance with a set protocol [9], as part of a broader study of tremor in MS. Only recordings from the dominant arm were used in this analysis, under the assumption that tremor in the non-dominant arm would not straightforwardly indicate functional disability. Each instance included three repetitions of the arm flexion-extension cycle, all of which were performed during verbal instruction and simultaneous demonstration by an experienced physiotherapist. The subjects were guided to move slowly, to prevent confusion caused by voluntary movements performed at tremor frequencies. The end-point of the movements (finger at nose) was sustained for a minimum of three seconds to allow a representative characterisation of the tremor. This movement stage was the focus of analyses in our study, because target-based movement is expected to evoke intention tremor, a particularly disabling form of tremor that is especially prevalent in MS.

**Fig. 2:**
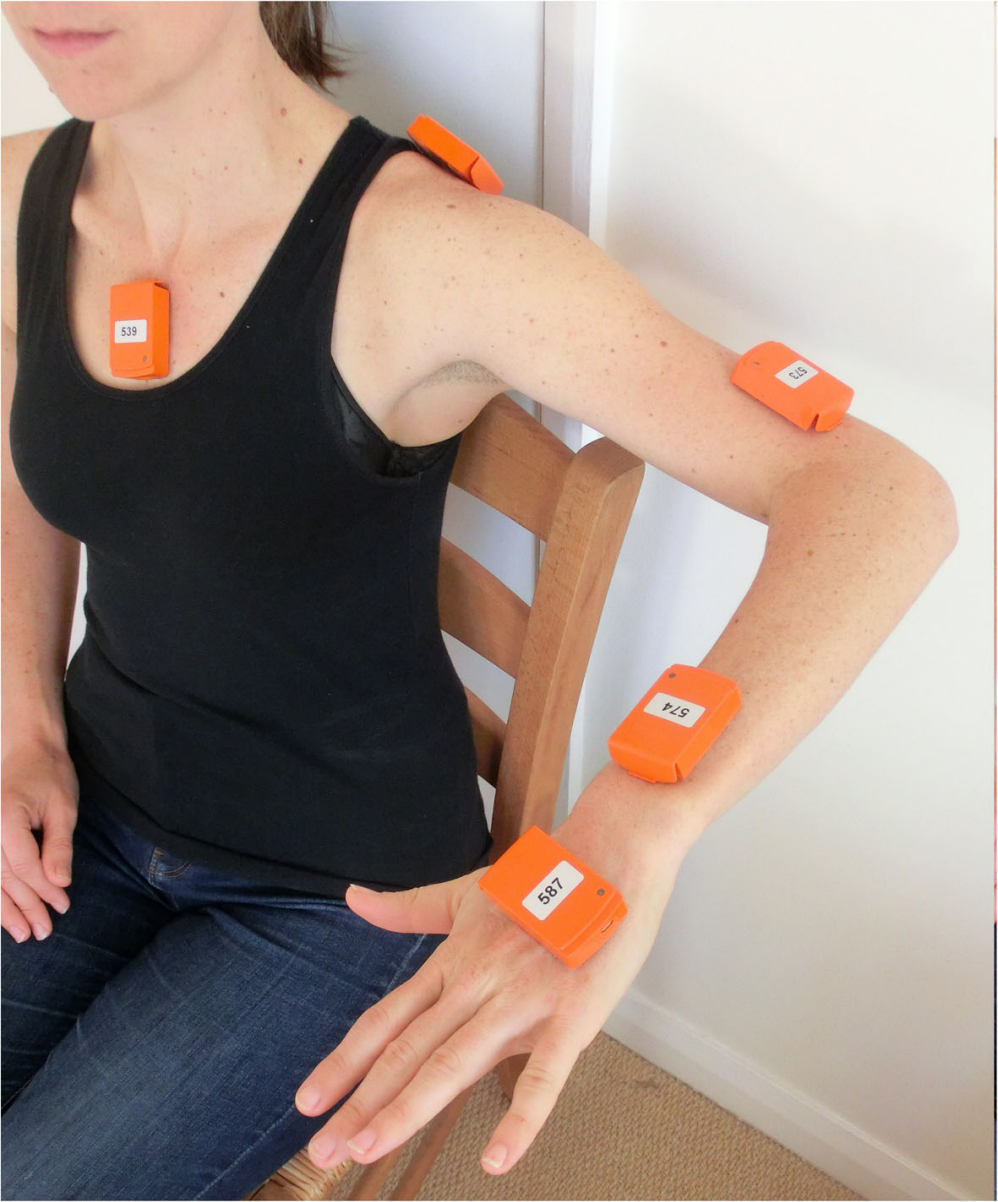
Photograph showing the positions of the five IMUs attached with double-sided adhesive

The subjects’ movements were recorded using a video camera and five IMUs (Xbus Kit and MTw Kit, Xsens Technologies B.V., Enschede, The Netherlands), as shown in Fig. 2.

At the start of each recording, the subject adopted a calibration pose for six seconds, avoiding sudden movements. This period allowed for settling of the manufacturer’s proprietary XKF-3 sensor fusion algorithm, based on the extended Kalman filter, which combined tri-axial accelerometer, gyroscope, and magnetometer readings to estimate the orientation of each IMU. A physiotherapist assisted them to hold the five body parts either parallel or at right angles to one another. At the end of this calibration period, the difference between the IMU orientation estimates and the idealised pose of the associated body segments were taken as fixed estimates of the IMUs’ relative alignments. Each IMU recording was synchronised with a video recording to aid interpretation of the data.

### Movement parameters

The IMU data were captured and analysed using our custom Tremtrace software, developed in MATLAB${\circledR }$. The movement stage of interest (fingertip within approximately 10 cm of the nose, neglecting involuntary excursions) was extracted manually. The analyst’s judgement was used to exclude any periods in which the subject deviated from the task in some way, such as by anchoring their fingertip on the nose rather than holding it floating in front of the nose as instructed.

From each instance of three repetitions, we sought to extract representative tremor amplitude estimates for each of fifteen joints, described previously in [17] and listed in Fig. 7.

The base of the subject’s torso was assumed to be fixed throughout each recording. Note that complex movements of the torso and shoulder girdle movement were simplified to three rotational joints each. Two joints, namely the elbow carrying angle and wrist pronation (distinguished from forearm pronation in being manifested more distally, between the hand and forearm IMUs), were not expected to exhibit substantial movement because they are not true anatomical joints. They were included for completeness of the kinematic reconstruction and to expose gross errors in sensor alignment.

In addition to rotations of the joints described above, we assessed translational and rotational movements of the hand, acknowledging the functional importance of this particular body segment’s motion as the cumulative result of the joint rotations. Hand displacements at tremor frequencies were calculated based on acceleration data from the hand IMU only. The accumulation of low-frequency drift due to integration of acceleration data was not problematic as frequencies below 2 Hz are not included in our analysis

Hand displacement and rotation were each considered only in the dominant tremor axis. The dominant translational and rotational tremor axes were identified separately for each movement stage by applying principal component analysis to the three-dimensional data (position or angular velocity in a global reference frame) after band-pass filtering at 2–10 Hz.

In summary, a total of seventeen movement parameters were considered: fifteen joint rotations, plus hand rotation and displacement in the dominant axes.

### Extraction of tremor amplitudes

For each window of interest in the recordings, we quantified tremor amplitudes from the original signals using the average tremor amplitude (ATA) [35].

8 $$  \textrm{ATA} = 4LT_{S}\sum\limits_{f=f_{1}}^{f_{2}}\sqrt{X(f)}  $$

*L* is the number of frequency bins in this tremor bandwidth, and *T*_*S*_ is the sampling period. *X*(*f*) is the unfiltered signal’s power spectral density, calculated by Welch’s method. The nominal tremor frequency *f*_*T*_ was identified by the maximum of *X*(*f*) within the 2–10 Hz bandwidth. To allow for a broad peak in *X*(*f*), the tremor bandwidth was identified by the boundary frequencies *f*_1_=*max*(2 Hz,*f*_*T*_−1 Hz) and *f*_2_=*min*(10 Hz,*f*_*T*_+1 Hz). The factor 4 is used so that, when the signal is a perfect sinusoid, ATA returns the peak-to-peak (rather than zero-to-peak) amplitude of that sinusoid, for a more intuitive representation of visual perceived amplitude.

Additional stages were included in our processing chain to emulate the common clinical practice of basing a tremor score on several repetitions of the task to improve the reliability of the score. Firstly, unreliable estimates were rejected using the heuristic conditions described in Appendix 1: conditions for rejecting tremor amplitude estimates. Subsequently, for each of the seventeen movement parameters, the surviving estimates of *log*(ATA) were averaged to form a representative estimate for that parameter in that instance of the task.

For all subsequent analyses, the measurement vectors were formed of some subset of these estimates.

### Measurement vectors and characteristic vectors

The subsets of movement parameters were chosen to focus on movement in either the hand or a selection of individual joints, as described in the following subsections.

#### Hand movement

Hand displacement and rotation in their respective dominant axes are two parameters that can be further combined into a more general description of hand movement. Clinical experience indicates that each individual’s tremor has its own characteristic blend of displacement and rotation produced at the hand. We quantified this blend by calculating a characteristic vector, using *j*=1 for hand displacement and *j*=2 for hand rotation, after normalisation as described in “Normalisation” section. The characteristic blend of hand displacement and rotation in a given limb was then taken as the two-element ‘characteristic vector’ calculated from Eq. 2 using these normalised values of *a*_*i,j*_.

#### Joint movement

Intuitively, the hand is a suitable point of measurement for capturing the functional impact of tremor. However, hand movement is an abstraction of individual joint movements, and important clinical information may be lost in that abstraction. The basic approach described above can be applied to any grouping of two or more individual joints to achieve an alternative tremor metric that may be more closely linked to the aetiology and anatomical distribution of the tremor, and thus more sensitive to disease modification/progression. In this case, z-score normalisation was not applied because the parameters were not heterogeneous, and such scaling would artificially inflate the significance of joints that do not tend to exhibit substantial tremor.

This approach can be applied across all recorded joints. However, it is conceivable that the inclusion of joints that do not consistently exhibit substantial tremor may introduce error to the measurement without incorporating useful information. A subset of joints can be selected, based on the ranking of their characteristic tremor amplitudes in that limb, but it is uncertain what number of joints would be optimal to include. This is one of the parameters explored in this study. All calculations of tremor in joint movement were repeated while varying *N*, the number of joints included in the calculation. In each case, only the *N* joints with the greatest values in the characteristic vector were included. Joints for which no value was returned due to exclusion of unreliable tremor estimates (see Appendix 1: conditions for rejecting tremor amplitude estimates) were placed at the bottom of this ranking.

For each class of measurement vector, each subject’s characteristic vector was calculated according to Eq. 2 based on all available recordings from that subject. Hence each characteristic vector was based on a maximum of four separate measurement vectors: two instances per visit, across two visits.

Changes in scale and profile were then calculated according to Eqs. 3 and 6, respectively.

### Alternative metrics for comparison

The primary aim of our analysis is to determine whether the proposed metrics, particularly change in scale, offers an improvement over conventional metrics in detecting clinically relevant changes in symptoms. Hence we must define these competing metrics as well as reference metrics against which to assess their efficacy.

#### Alternative sensor-Based metrics

The most obvious sensor-based metric for a change in tremor symptoms is the simple difference in a univariate tremor amplitude estimate. Our analysis includes hand displacement, hand rotation, and rotation in the joint with the greatest tremor amplitude as examples of such univariate metrics.

For multivariate tremor measurements, such as those described in “Measurement vectors and characteristic vectors” section, the mean difference may be taken as a simple difference metric. We define the mean difference, *d*_*M*_, as the mean, over all movement parameters considered, of the difference between two measurement vectors.

9 $$  d_{M}\left(\boldsymbol{a_{A}},\boldsymbol{a_{B}}\right) = \frac{\sum\nolimits_{j \in W_{A,B}}\left(a_{B,j}-a_{A,j} \right)}{n_{A,B}}  $$

*W*_*A,B*_ is the set of *n*_*A,B*_ movement parameter indices (*j*-values) for which *a*_*A,j*_ and *a*_*B,j*_ are both non-null.

#### Metrics from a conventional clinical scale

As noted by the MDS task force [14], sensor-based metrics have not yet been shown to offer any practical benefit over conventional scales in detecting clinically relevant changes. Hence we included metrics from a conventional clinical scale in our comparisons.

The FTMTRS [8] is the most widely used clinical scale for the assessment of tremor in MS [20]. It consists of three parts, each containing multiple components scored on an integer scale from 0 (least severe) to 4 (most severe).

In Part A, the examiner rates the severity of the subject’s resting, postural, and action/intention tremor in various body parts. In this study, we have focussed strictly on the dominant-limb intention tremor component of Part A, because this tremor category is considered to be particularly prevalent and debilitating in MS. This score is henceforth denoted ‘FTMTRS A’. Like the sensor-based measurements, it was based on the subject’s performance of a finger-to-nose test. Scoring was performed by a single, trained, experienced observer (an MS-specialist physiotherapist). Previous studies have shown FTMTRS A to have good intrarater repeatability (Spearman’s *ρ*≥0.92 [15, 32]) and moderate/good interrater repeatability (Spearman’s *ρ*≥0.73 [15, 32], Cohen’s *κ*≥0.54 [9, 32]). The rater in our study participated as a rater in [9]. Thus the inter-rater reliability of her scoring has been validated.

In Part B, the subject’s functional disability is rated in a standardised manner according to their performance in handwriting, three drawing tasks, and a water pouring task. In this study, ‘FTMTRS B’ was taken as the sum of the scores achieved with the dominant hand in these tasks.

Part C of the FTMTRS is the subject’s self-assessment of their tremor’s functional impact on several activities of daily living: speaking, feeding, drinking, hygiene, dressing, writing, and working. We denote the sum of these scores, excluding speaking, as ‘FTMTRS C’.

Note that controlling for intrinsic variability in the manner described in “Controlling for intrinsic variability” section is not pragmatic for metrics based on the FTMTRS; same-day differences are expected to be zero due to the coarseness of these scales, and the result of Eq. 7 would thus be undefined.

#### Reference metrics

Previous studies [6, 14, 23] have compared tremor metrics on the basis of minimum detectable change (MDC). We chose instead to examine and compare the efficacy of various tremor amplitude metrics in tracking changes in reference metrics representing functional disability. MDC cannot straightforwardly be applied to metrics of change that are measured in an entirely different direction from the absolute metric. Furthermore, the validity of MDC is dependent on assumptions about the statistical distribution of the metric. In contrast, our use of Spearman rank correlations does not rely on assumptions about the data distribution, ensuring a fair comparison between very different metrics. Having acknowledged the multidimensional nature of tremor, evaluating the metrics based on correlations with measures of functional disability ensures that we identify metrics with clinical relevance, rather than favouring a metric that is repeatable but insensitive to the most important aspects of the tremor.

The tremor amplitude metrics considered comprise all aforementioned sensor-based metrics along with FTMTRS A. Our primary measurement of functional disability is FTMTRS C, because it captures a subject’s symptoms and their impact beyond the limit of what may be exposed during the clinical visit. The subjective nature of this score is a notable limitation when comparing individuals, although it is unlikely that the subject-specific biases would correlate systematically with errors in the sensor-based tremor amplitude measurements. In other words, while the subjective assessment of the tremor’s impact may be a ‘noisy’ measure of the tremor’s ‘true’ impact, when measuring intra-individual changes a sensor-based metric that exhibits statistically significant correlation in spite of this noise can reasonably be interpreted as capturing meaningful changes in the impact of tremor.

Nonetheless, to avoid solely depending on a subjective reference metric we include FTMTRS B in our analyses as a more objective, albeit less comprehensive, measure of functional disability.

### Quantifying the amplitude of a single multivariate tremor measurement

Although our primary focus is on the efficacy of different metrics in detecting changes in symptoms, our understanding of these metrics may be improved by examining the direct relationships between single measurements of different types. To enable the comparison of multi-dimensional tremor measurements with individual FTMTRS C scores, we must extract a single value representing the tremor severity in that multi-dimensional measurement. For this purpose, we quantified the tremor severity in any measurement vector using the mean of its non-null components, $\overline {a_{i}}$.

10 $$  \overline{a_{i}} = \frac{\sum\nolimits_{j \in W_{i}}{a_{i,j}}}{n_{i}}  $$

*W*_*i*_ is the set of movement parameter indices (*j*-values) for which *a*_*i,j*_ is non-null in the *i*^th^ measurement. *n*_*i*_ is the number of elements in *W*_*i*_.

### Statistical analyses

The relationships between tremor amplitude metrics and reference metrics were examined based on Spearman’s rank correlation coefficient to avoid excessive influence from outliers and avoid assumptions about the distribution of the data. Statistical significance of these correlations was determined based on p-values calculated using MATLAB${\circledR }$’s corr function, using a threshold of *p*<0.05.

For joint movement metrics, multiple variants were considered for 1<*N*≤15 (i.e. multiple comparisons were made), presenting a risk of Type I statistical errors. To address this concern, we applied the Benjamini-Hochberg (BH) procedure [3] to this family of results to control the false discovery rate (FDR). The estimated false discovery rate for each correlation, *pFDR*, was used as a conservative replacement for the p-value and set against the same threshold. Note that the BH procedure is conservative when the candidate metrics can be assumed to be positively correlated with each other, as is the case here.

## Results

### Spearman rank correlations

**Fig. 3:**
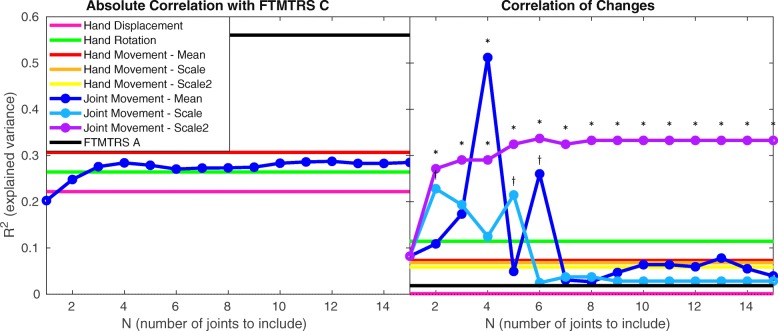
Left panel: Squared Spearman’s rank correlation coefficients comparing tremor amplitude metrics against FTMTRS C (self-assessed impact of tremor). The parameter *N* applies only to joint movement, hence all other metrics are represented by horizontal lines. All correlations in this panel are statistically significant. The legend applies to both panels, although the ’change in scale’ metrics (see Eqs. 3 and 5) feature only in the right panel. Right panel: As in the left panel, but comparing the tremor amplitude *change* metrics, controlling for intrinsic variability (see Eq. 7) against the *change* in FTMTRS C. ∗ denotes statistical significance at *pFDR*<0.05. *†* denotes that the result does not survive false discovery rate control, i.e. *p*<0.05 and *pFDR*≥0.05

Figure 3 depicts the correlations between tremor amplitude metrics and subjects’ self-assessment of the impact of their tremor on activities of daily living (FTMTRS C). For each correlation, at least 15 data points were available (15 or more subjects with valid measurements on two different days, and two valid same-day measurements for at least one of those days). As shown in the left panel, clinical observation (FTMTRS A) exhibited substantially stronger correlation than all sensor-based metrics, although every amplitude metric proved to be a significant correlate. This indicates that FTMTRS A more accurately captures an individual’s tremor severity relative to the broader population.

However, as depicted in the right panel of Fig. 3, FTMTRS A and all sensor-based metrics restricted to the hand exhibited no significant correlation with the subjects’ change in self-assessed tremor impact between visits. In contrast, several variants of the joint movement metrics exhibited statistically significant correlations with this change. The scale2 metric (5) exhibits statistical significance for all *N* values (except the trivial case of *N*=1, at which scale changes are effectively identical to the mean change). The strongest correlation is exhibited by the mean change for the case in which only the four most tremulous joints are considered (*N*=4). However, the plots for mean change and scale change peak erratically in the range 1<*N*<7, beyond which they exhibit no substantial correlation (*R*^2^<0.1). These results suggest a particular strength of the scale2 metric; by exaggerating the influence of the most tremulous joints on a continuous scale, it reduces the dependence of the number of joints included.

**Fig. 4:**
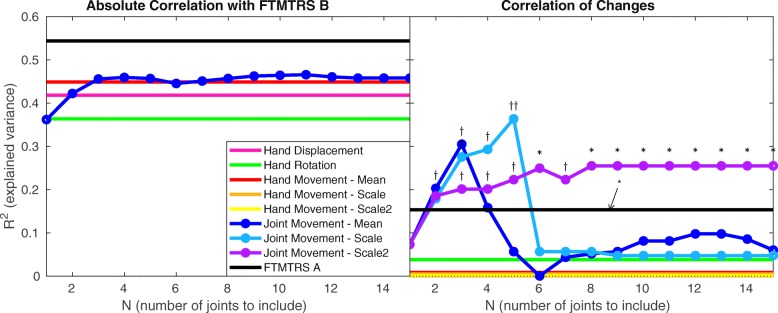
Similar to Fig. 3, but comparing tremor amplitude metrics against FTMTRS B (task-based assessment of functional disability). Again, all correlations represented in the left panel are statistically significant. In the right panel, *††* denotes that *p*<0.01 but *pFDR*≥0.05 (result rejected by false discovery rate control)

Figure 4 presents equivalent correlations against our second reference metric, task-based assessment of functional disability (FTMTRS B). Again all tremor amplitude metrics exhibit statistically significant absolute correlation, and FTMTRS A is more strongly correlated than the sensor-based metrics, albeit by a reduced margin in this case (left panel). As seen in the right panel, the scale2 metric achieves statistical significance at large values of *N* (*N*=6 and *N*>7). FTMTRS A also exhibits statistical significance in this case, albeit with a lesser *R*^2^ value. None of the sensor-based change metrics restricted to the hand exhibit statistically significant correlations.

The mean and scale change in joint movement present the strongest correlations with change in FTMTRS B, in the range 2<*N*<6. These results are not regarded as statistically significant; they do not survive false discovery rate control, largely due to the weakness of correlations beyond *N*=6. Nonetheless, the pattern of these plots combined with the results for scale2, here and in Fig. 3, suggests that these metrics may have a genuine sensitivity to change in functional disability over a limited range of *N* values. Fresh data would be required to test this suggestion rigorously and explore the possibility that, with more informed parameter selection, the scale metric may be superior to the scale2 metric. From the available data, the evidence that scale2 has merit is far more compelling.

### Individual level data

In this section, we present more detailed results from the FTMTRS metrics. It is not feasible to present detailed results from all the variants of sensor-based metrics, but we present those for scale2 (with *N*=15), which was arguably the most promising new metric. As noted in the previous section, the available data allowed scale2 to be calculated for fifteen subjects; two did not return, three did not record from their dominant arm on the second visit, and in a further four tremor was not reliably detected on at least one of the days. The characteristics of this sub-cohort are summarised in Table 2. Similar to the full cohort, paired t-tests revealed significant change in FTMTRS C (*p*=0.010), but not FTMTRS A and FTMTRS B (*p*=0.38 and *p*=0.13, respectively). Additionally, scale2 exhibited a marginally significant increase (*p*=0.054).

**Table 2:** Summary for Sub-Cohort from which Scale2 was Calculated

		FTMTRS - Visit 1			FTMTRS - Visit 2			
	age	A	B	C	A	B	C	Days
mean	47	1.6	6.6	14.6	1.5	7.6	16.4*	153.1
min	30	0	1	6	0	0	9	17
max	67	4	20	24	4	20	24	267

^*^Significant change from visit 1; *p*<0.05

**Fig. 5:**
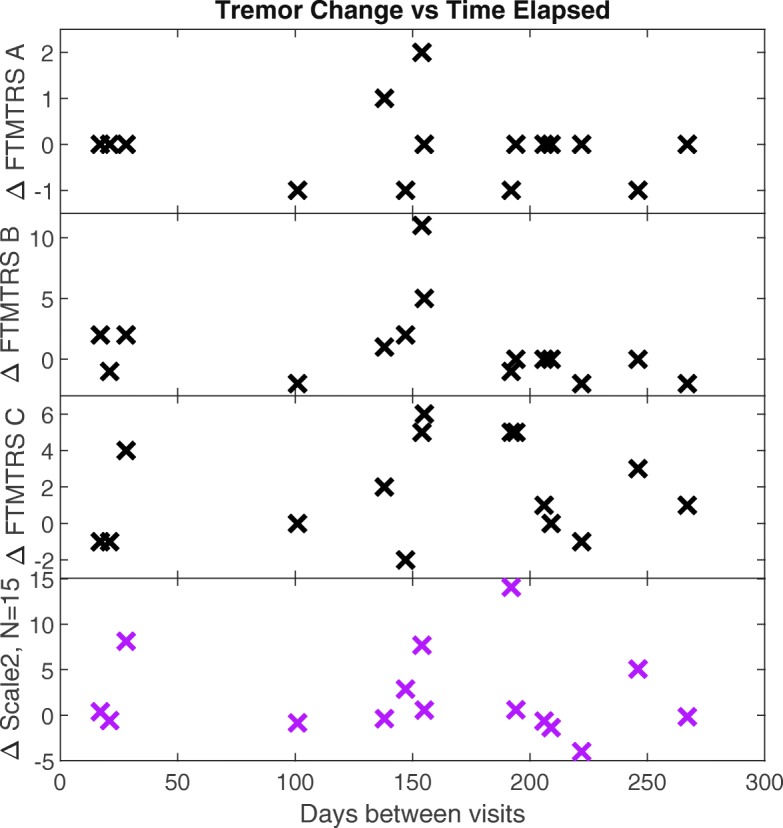
Relationship between tremor metrics and time for the fifteen individuals for whom scale2 could be calculated

Figure 5 shows the change in tremor versus time for scale2 at *N*=15 and for the three FTMTRS metrics. None of the metrics exhibited a significant correlation with the number of days between visits. This reflects the highly variable nature of disease progression in MS.

**Fig. 6:**
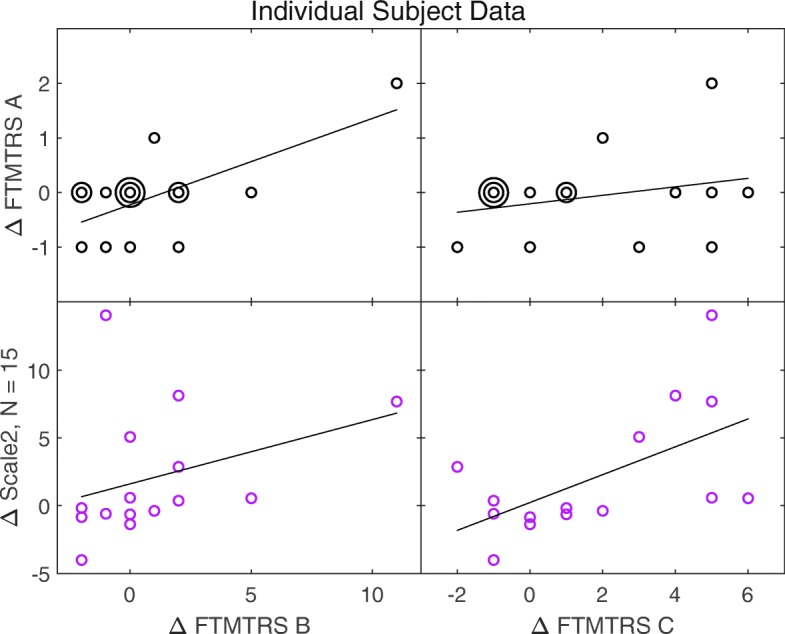
Changes in tremor metrics for individual subjects, superimposed with the line of best fit for each pairing. Where data points coincide exactly, the number of coincident points is represented by the number of concentric circles. The fitted lines should not be interpreted as the basis for the correlations reported in Figs. 4 and 3, which were calculated as Spearman rank correlations, rather than linear ones

Figure 6 compares the changes in tremor metrics for individual subjects. While some extreme data points can be seen, the use of Spearman’s rank correlation as the basis for comparison ensured that these did not exert undue influence.

**Fig. 7:**
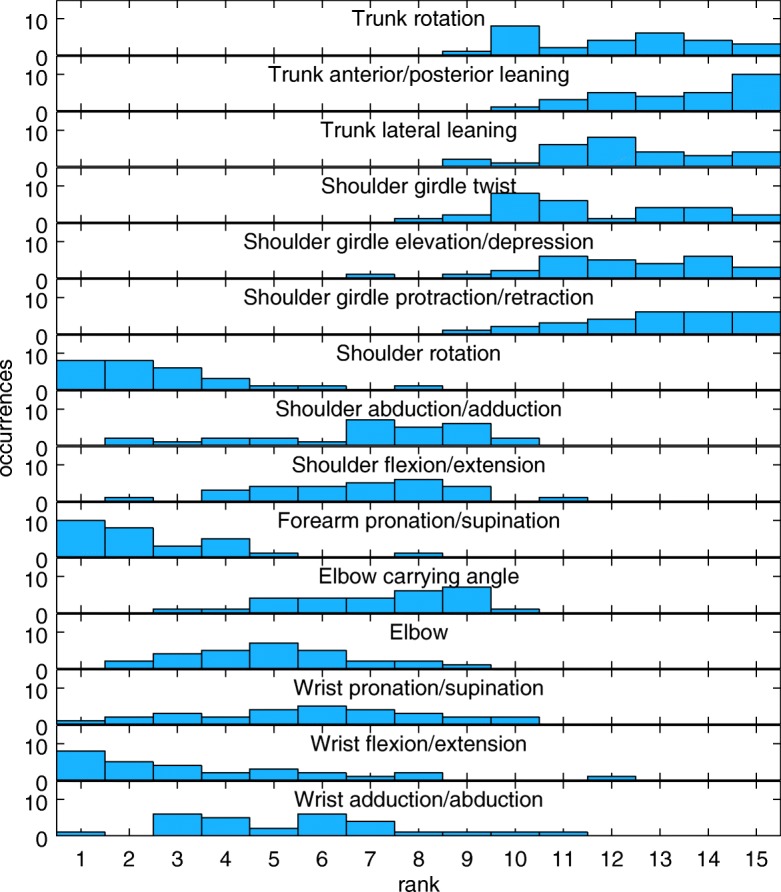
Histograms of each joint’s rank within the characteristic vector of each of the 24 limbs analysed. ‘Occurrences’ on the vertical axis, is the number of limbs in which that joint achieved a particular rank. A rank of 1 implies that this joint had the greatest tremor amplitude in that limb

### Dominant joints

Given that the discriminatory power of the joint movement metrics was found to be sensitive to the number of joints included, it is important to consider whether any joints were preferentially included or excluded in these calculations. Figure 7 presents histograms of each joint’s rank within the characteristic vector of each of the 46 limbs recorded. We see that, shoulder rotation, forearm pronation/supination, and wrist flexion/extension are each among the three joints of greatest tremor amplitude in the vast majority of limbs. The most proximal joints in our model, those of the shoulder girdle and trunk, presented the six lowest tremor amplitudes (ranks 10–15) in almost every limb.

It is important to note that these distributions of joint involvement are likely to be specific to the task and pathology (MS) considered in this study. Furthermore, the ranking of joints according to the amplitude of rotation should not be interpreted as a reliable ranking of their functional impacts.

## Discussion

The results presented confirm that the power of sensor-based metrics to discern clinically relevant changes in MS tremor symptoms can be improved by using personalised tremor profiling. This improvement yields statistically significant correlates of changes in functional disability and self-assessed impact of tremor on daily living, outperforming conventional clinical observations in these regards. Thus the newly proposed metrics overcome the perceived limitations of sensor-based tremor assessments identified by the MDS taskforce [14].

### Comparison with conventional practice

These results demonstrate the potential importance of sensor-based tremor measurements as a complement to subjective and task-based scales in monitoring changes in an individual’s symptoms, e.g. during clinical trials, personalised assessments of treatment efficacy, or routine clinical visits. Several variants of the newly proposed metrics outperformed FTMTRS A as a correlate of change in functional disability or tremor impact.

Conversely, FTMTRS A outperformed all sensor-based metrics in terms of direct correlations with the reference metrics. A likely contributing factor is the fact that the sensor configuration used in this study does not capture all aspects of limb movement. Most notably, movement of the fingers was not captured, although such an extension of our method is technically feasible with optical motion tracking systems or with recently developed commercial IMU systems such as Noitom Perception Neuron${\circledR }$. In applying the FTMTRS A score, the clinician is able to account for all visible aspects of limb movement. Furthermore, the scale does not specify exactly which part of the hand should be observed. This allows the clinician to adapt their point-of-interest, consciously or unconsciously, to account for the likely functional impact of the observed symptoms. The clinician may also take into account other sources of information, such as vocal cues, facial expression, or awareness of the patient’s movement characteristics outside the period of interest, to distinguish between voluntary and involuntary movement and to discount events that are not representative of the subject’s symptoms. Although our sensor-based approach allows the analyst to implement such decision-making when extracting the movement stages of interest, this influence is inevitably constrained by the limitations of the software interface.

The overall richness of information and clinical judgement contributing to the FTMTRS A score explains the primacy of this metric as a single-measurement indicator of functional disability. However, while this rich input informs the FTMTRS A score, it is not captured for future reference, hence these benefits are lost when comparing two measurements. Furthermore, the coarse resolution of the FTMTRS A, a necessity for intra- and inter-rater consistency, limits the scale’s sensitivity to change. The dependence of sensitivity on resolution is demonstrated more explicitly in Appendix 2: the effect of scale discretisation on correlation, using simple numerical experiments. The extreme weakness of the correlation between changes in FTMTRS A and FTMTRS C may be surprising, given the relatively strong absolute correlation. However, our result here is in agreement with van der Stouwe et al. [33], who found that changes in FTMTRS C were significantly correlated with changes in FTMTRS B, but not FTMTRS A. In summary, the precision, objectivity, and rich data storage afforded by sensor-based metrics are advantageous in identifying changes in symptoms, as our study demonstrates.

It might be argued that a fairer comparison of observational and sensor-based metrics would be one in which the clinician identifies a change in symptoms by directly comparing video recordings between sessions. However, in clinical practice this approach would consume considerably more of the clinician’s time. Furthermore, no validated scales exist to guide the scoring of such comparisons, and we cannot envisage a straightforward manner in which such a scale would standardise the incorporation of more than two recording sessions. In contrast, the extraction of sensor-based metrics comparing any number of available recordings requires no appreciable increase in effort from the clinician.

### Physiological interpretation

The newly proposed metrics defy the notion that tremor in MS is too variable to justify attempting to measure it with greater precision than that afforded by conventional scales [14]. It appears that the calculation of the characteristic vector allows us to ascribe some regularity to the tremor, even when its amplitude is not regular. Effectively, deviations from this profile are then identified and discarded, removing a source of variance from the measurement.

Although our approach has been shown to reveal clinically relevant changes, it would be rash to assume the discarded components are clinically irrelevant. MS is a progressive condition starting in most individuals with a relapsing remitting phase but transforming over time to a secondary progressive phase with few or no relapses. This later phase is characterised by increasing disability mostly without evidence of new plaque formation. Most patients seen in this study were in the secondary progressive phase and sudden acute changes would not be expected. Where relapses do occur, tremor changes are perhaps more likely to manifest as a change in profile rather than scale, due to the formation of new plaques. A future area of interest would be to confirm whether progressive and relapsing remitting symptom changes tend to manifest separately in scale and profile changes, respectively.

### Refinements and variations of our approach

Our study tested several different implementations of the principle of personalised tremor profiling, and there are several other ways in which the implementation of our method could vary.

We have demonstrated that personalised tremor profiling allows perceptible change in the tremor’s functional impact (FTMTRS C) to be identified. Although the mean change in joint movement (without profiling) achieved stronger correlations than the scale metrics in isolated cases, it showed strong sensitivity to the number of joints included, *N*, leaving no clear optimal choice for this parameter. The proposed use of a characteristic vector appears to reduce this sensitivity to *N*, especially in the case of the scale2 metric, which allows a conservatively high value of *N* to be chosen without loss of efficacy. It is likely that none of the considered variants of scale change represents the optimal use of tremor profiling. In particular, there are many conceivable variants of tuning the characteristic vector or the scale change calculation (i.e. alternatives to Eqs. 4 and 5), which have yet to be explored. Nonetheless, our study provides proof-of-concept that such an approach can yield a metric that is superior to coarse-scaled visual observation in indentifying clinically relevant changes in symptoms.

The distinction between hand and joint movement warrants attention, not least for reasons of practicality. Hand movement may be recorded with a single sensor unit, or even with standard smartphones or smartwatches [19], making it particularly well suited to frequent at-home monitoring. This convenience could conceivably allow closer clinical monitoring without substantially increasing the burden on the patient, and our approach could easily be incorporated into those systems. Our results indicate that, in terms of absolute correlations, the combined hand movement metrics yield a slight improvement on the use of displacement or rotation alone. Notably, however, our newly proposed hand movement metrics were not found to improve on simple hand displacement/rotation measurements in terms of sensitivity to *changes* in symptoms in these single sensor configurations.

For all joint movement metrics, no substantial improvement in performance was achieved beyond *N*=6. Considering the joint rankings presented in Fig. 7, this suggests that movements within the torso (trunk and shoulder girdle) do not offer useful new information on functional disability or the impact of tremor. One may therefore be tempted to seek reduced sensor configurations for convenience. However, shoulder rotation presented as one of the three most tremulous joints in the vast majority of cases. Hence we advise that some sensing of the torso is necessary, even if it is only compared with the upper arm in order to identify shoulder movement.

Individual finger movements are also expected to yield relevant information, especially when manual dexterity is impaired. Finger movements were not captured in our study, but our analytic method is suited to such variations.

Our study focussed on recordings captured during a very specific task, in which the subject attempts to hold their finger just in front of their nose. This task was chosen because it is commonly used in clinical tremor assessments and is expected to evoke intention tremor, which is believed to be particularly disabling. Further study is required to examine the extent to which multi-joint tremor profiling may be usefully applied to other tasks and tremor types. Although these other embodiments of the proposed metrics would not be expected to correlate as strongly with disability changes, they may still be useful biomarkers of disease progression.

Alternative implementations of our method may also make use of different movement parameters from those considered here. For example, joint torques estimated by inverse dynamics [17] or individual joint contributions to hand velocity/acceleration [26] may be more functionally relevant than joint rotations. Furthermore, the characteristic vector may incorporate the frequency and/or relative phase of the oscillations in individual movement parameters, rather than just the amplitude. We intend to explore such variants of our approach in future work.

A notable limitation of our sensor-based approach is that it does not robustly distinguish ‘true’ tremor from ataxic movements. The same may be true of FTMTRS B and FTMTRS C. Therefore, hypothetically, a strong common contribution of ataxia might exaggerate the efficacy of the proposed metrics as indicators of tremor. Visual observation (i.e. FTMTRS A) may well be superior in this regard, drawing on the skill and experience of the observer, but it is a challenging task in any case. The strong absolute correlation between FTMTRS A and FTMTRS B/C suggests that ataxic contributions do not dominate. Future work may be directed at developing algorithms that objectively discriminate between tremor and more general ataxia. Such an algorithm could conceivably be appended to the beginning of our processing chain to eliminate the influence of ataxia on the tremor measurements. The importance of this step would depend on the nature of the treatment under consideration and whether it selectively targets either tremor or ataxia.

The data processing techniques used in later stages of our method also present scope for variation. In particular, there may be many other valid ways in which to calculate the characteristic vector that serves as our personalised tremor profile. Our method discards phase information, but the relative phase of tremor in different movement parameters may be an important aspect of an individual’s tremor profile, as may the frequency of the tremor. However, such features are likely to have a complex, non-monotonic relationship with the tremor’s perceived or functional impact, making our simple subtractive definitions of change in scale (Eq. 3) inappropriate. Machine learning techniques such as neural networks may be better suited to accommodating such complexities. We have avoided such techniques in this study because the known inter-individual heterogeneity of MS tremor symptoms would present substantial risk of over-fitting, especially in a relatively small cohort such as ours. However, our study provides proof-of-principle that the inherent variability of MS tremor symptoms can be overcome with appropriate data analysis to achieve sensor-based metrics that capture changes in functional disability (self-perceived and objectively measured) more accurately than subjective observation.

## Conclusions

Personalised multi-joint tremor profiling allows clinically relevant changes in symptoms to be exposed in objective, precise, detailed movement recordings. The newly proposed metric based on a squared characteristic vector was observed to show statistically sound correlation with changes in the functional impact of tremor, whether based on self-assessment (FTMTRS C) or task performance (FTMTRS B). Contrary to previous studies, we have shown that sensor-based measurements can improve on conventional clinical observation (FTMTRS A) in terms of sensitivity for this task.

There are multiple ways in which this improvement can be expected to benefit people with tremor. Personalised treatment selection may be improved by more accurately characterising the effects of a newly prescribed treatment, especially in MS where movement disorders are complex and subject to change. More broadly, convenient in-home monitoring could allow data on symptom developments to be captured in detail for fast, well-informed clinical intervention. Finally, the development of new treatments may be accelerated by allowing clinical trials to acheive adequate statistical power in shorter or smaller studies, thus reducing practical or financial cost as deterrents to research.

Future investigations will explore how the method may be refined and extended to other movement tasks and tremor categories.

## Appendix 1: conditions for rejecting tremor amplitude estimates

To prevent the analyses from being distorted by unreliable amplitude estimates, ambiguous scenarios were automatically identified and excluded according to the rules described below. These rules were developed and tuned heuristically according to the authors’ experience and judgement in comparing tremor estimates to video recordings to ascertain confidence in the estimate.

Firstly, the amplitude estimate was rejected if the signal section isolated by the user was shorter than 1.5 s, which would correspond to three full cycles at 2 Hz, the lowest possible tremor frequency considered. Although the 1.5-s threshold defies the conventional guidance that the window length for Fourier analysis should contain at least 10 full cycles at the lowest frequency of interest to allow accurate characterisation, this relaxation of the rule was deemed acceptable for the following reasons: 1) the extreme case of 2 Hz tremor would not occur frequently; 2) fine frequency resolution was not required; 3) inaccuracies in amplitude estimates would be attenuated by averaging across three repetitions of the task; and 4) ambiguities attributable to the use of a short analysis window would be excluded by further criteria, described in the next paragraphs. In current practice, clinical assessments of complex tremor cases are often made on the basis of multiple short bursts of a few tremor cycles, particularly when the tremor is highly intermittent. Our approach reflects this practice.

The minima within 1.5 Hz either side of the peak in *X*(*f*) were taken as conservatively wide bounds of the tremor band. The array of frequency bins included in the tremor band is denoted ***f***_***TB***_. The remainder of the 2–10 Hz band, denoted ***f***_***CB***_, was used as a basis for comparison. To avoid ambiguities between tremor and broadband noise, which might arise from sensor noise or from non-periodic movement, the amplitude estimate was rejected if the average power in the tremor band was less than the median (50^th^ percentile) of power in the comparison band, which can be written as follows

11 $$  \overline{ X(\boldsymbol{f_{{TB}}})} < P_{50}(X(\boldsymbol{f_{{CB}}}))  $$

The bar notation on the left hand side of this inequality denotes the mean of that series. If inequality 11 was satisfied, further criteria were examined to avoid ambiguous cases in which the suspected tremor peak was actually one of many similarly large peaks in the spectrum, which might be caused by complex movements, spectral leakage, or harmonics of the short Fourier window. Non-rectangular windowing functions are conventionally used to reduce the occurrence of such ambiguities, but this approach was considered unattractive in our application due to the extremely short signal lengths considered. Instead of prevention, we chose a strategy of exclusion. The array of differences between consecutive local extrema of *X*(***f***_***CB***_), denoted ***Δ***_***CB***_, was compared against the range of *X*(***f***_***TB***_),*Δ*_*TB*_.

12 $$ \Delta_{{TB}}=max(X(\boldsymbol{f_{{TB}}}))-min(X(\boldsymbol{f_{{TB}}}))  $$

The amplitude estimate was rejected if the power range of ***f***_***TB***_ was small compared to the fluctuations in ***f***_***CB***_ or, more specifically, if either of the following conditions were satisfied.

13 $$ \Delta_{{TB}} < 2P_{70}(\boldsymbol{\Delta_{{CB}}})  $$

14 $$ \Delta_{{TB}} < \overline{ \boldsymbol{\Delta_{{CB}}}} + 3\sigma(\boldsymbol{\Delta_{{CB}}})  $$

where *σ*(***Δ***_***CB***_) denotes the standard deviation of ***Δ***_***CB***_.

## Appendix 2: the effect of scale discretisation on correlation

Previous authors have noted that the coarse resolution of five-point tremor scales such as FTMTRS A makes them insensitive to change [7, 33]. While this is easily understood when considering whether changes in a single patient will be detected, it is less obvious how the effect manifests in a collective analysis such as the Spearman rank correlation. To illustrate the effect of discretisation (i.e. using a coarse scale) on correlation coefficients, we conducted two simple numerical experiments.

**Fig. 8:**
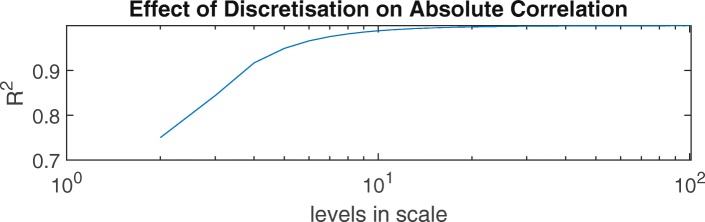
Discretising a scale reduces the Spearman rank correlation from *R*^2^=1 in the continuous case to *R*^2^=0.75 in the extreme case of a scale with just two distinct levels

Firstly, focussing on absolute correlations, we generated a set of 100,000 *x*-values randomly from a continuous, uniform distribution. We copied these values to form a perfectly correlated set of *y*-values, which were then rounded to a given number of levels. Figure 8 shows that as the number of levels decreased from 100 to 2, the Spearman rank correlation between *x* and discretised *y* increasingly deviated from 1. However, the effect is relatively small, with *R*^2^=0.75 for the extreme case of just two levels in the scale.

**Fig. 9:**
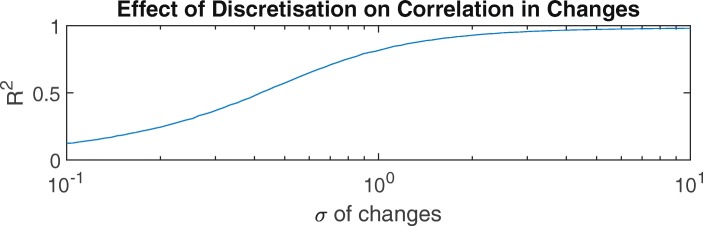
In a discretised scale, the correlation of changes with changes in another scale depends on the magnitude of changes relative to the scale interval. *σ* refers to the standard deviation of a Gaussian distribution with mean of zero. *R*^2^ is the explained variance, based on Spearman rank correlation

In the second experiment, we explored the effect of correlations between changes in values. We fixed the range of the scale at 0–4 and rounded *y* to integer values, matching FTMTRSA. We then generated a set of random variations, *Δx*, with zero-mean Gaussian distribution and a standard deviation *σ* that varied from 0.1 to 10. The same variations *Δx* were applied to *x* and the continuous *y*-values, before rounding was applied to these shifted *y*-values. *Δy* was then calculated by subtracting these rounded, shifted values from the originals. Figure 9 shows that when the changes were large relative to the scale interval (1), the correlation coefficient between *Δx* and *Δy* was close to 1, but when the average change was smaller than the scale interval, as for our dataset, *R*^2^ was substantially reduced.

## Data Availability

The data collected and analysed in the current study, along with the analysis code, are available in the Open Science Framework repository: https://osf.io/df3bt/

## Abbreviations

ATA: Average tremor amplitude
FTMTRS: Fahn-Tolosa-Marin tremor rating scale
FTMTRS A: The score (0–4) given by a clinician to describe a subject’s intention tremor, based on visual observation of a finger-to-nose task performed with the dominant limb
FTMTRS B: The summed score (0–20) describing a subject’s performance in five functional tasks (writing, 3 ×drawing, and water pouring)
FTMTRS C: The sum (0–24) of self-assessed scores given by a subject to quantify the impact of their tremor on their functional ability in six general activities of daily living: feeding, drinking, hygiene, dressing, writing, and working
IMU: Inertial measurement unit
MDS: Movement disorder society
MS: Multiple sclerosis
